# The *Cryptosporidium parvum *Kinome

**DOI:** 10.1186/1471-2164-12-478

**Published:** 2011-09-30

**Authors:** Jennifer D Artz, Amy K Wernimont, Abdellah Allali-Hassani, Yong Zhao, Mehrnaz Amani, Yu-Hui Lin, Guillermo Senisterra, Gregory A Wasney, Oleg Fedorov, Oliver King, Annette Roos, Vlad V Lunin, Wei Qiu, Patrick Finerty, Ashley Hutchinson, Irene Chau, Frank von Delft, Farrell MacKenzie, Jocelyne Lew, Ivona Kozieradzki, Masoud Vedadi, Matthieu Schapira, Chao Zhang, Kevan Shokat, Tom Heightman, Raymond Hui

**Affiliations:** 1 Structural Genomics Consortium, University of Toronto, MaRS South Tower, Floor 7, 101 College St, Toronto, Ontario M5G 1L7, Canada; 2 Structural Genomics Consortium, University of Oxford, Old Road Research Building, Roosevelt Drive, Oxford OX3 7DQ, UK; 3 Howard Hughes Medical Institute, Department of Cellular and Molecular Pharmacology, University of California at San Francisco, San Francisco, California 94158, USA

## Abstract

**Background:**

Hundreds of millions of people are infected with cryptosporidiosis annually, with immunocompromised individuals suffering debilitating symptoms and children in socioeconomically challenged regions at risk of repeated infections. There is currently no effective drug available. In order to facilitate the pursuit of anti-cryptosporidiosis targets and compounds, our study spans the classification of the *Cryptosporidium parvum *kinome and the structural and biochemical characterization of representatives from the CDPK family and a MAP kinase.

**Results:**

The *C*. *parvum *kinome comprises over 70 members, some of which may be promising drug targets. These *C. parvum *protein kinases include members in the AGC, Atypical, CaMK, CK1, CMGC, and TKL groups; however, almost 35% could only be classified as OPK (other protein kinases). In addition, about 25% of the kinases identified did not have any known orthologues outside of *Cryptosporidium spp*. Comparison of specific kinases with their *Plasmodium falciparum *and *Toxoplasma gondii *orthologues revealed some distinct characteristics within the *C. parvum *kinome, including potential targets and opportunities for drug design. Structural and biochemical analysis of 4 representatives of the CaMK group and a MAP kinase confirms features that may be exploited in inhibitor design. Indeed, screening *Cp*CDPK1 against a library of kinase inhibitors yielded a set of the pyrazolopyrimidine derivatives (PP1-derivatives) with IC_50 _values of < 10 nM. The binding of a PP1-derivative is further described by an inhibitor-bound crystal structure of *Cp*CDPK1. In addition, structural analysis of *Cp*CDPK4 identified an unprecedented Zn-finger within the CDPK kinase domain that may have implications for its regulation.

**Conclusions:**

Identification and comparison of the *C. parvum *protein kinases against other parasitic kinases shows how orthologue- and family-based research can be used to facilitate characterization of promising drug targets and the search for new drugs.

## Background

More than 58 million children are afflicted annually with diarrheal disease associated with the most prevalent infections of the small intestine, including *Escherichia coli*, *Rotavirus*, *Giardia lamblia*, and *Cryptosporidium parvum*, which ultimately results in the death of 2.5 million children [1]. *C. parvum *is an obligate parasite in the same phylum of *Apicomplexa *as *Plasmodium *and the same order of *Eucoccidiorida *as *Toxoplasma *and *Eimeria*. It is one of the pathogenic agents responsible for cryptosporidiosis, a zoonotic and enteric disease. Children in resource-poor settings are particularly at risk, not only with an increased incidence of *Cryptosporidium spp*. infection, but also with increased acute and long-lasting morbidity. Psychomotor developmental stunting may occur following infection, especially in children under one year of age, with its effects still measurable many years after infection [2,3]. Malnutrition is both a contributing factor and a result of *Cryptosporidium spp*. infection [4,5]. In this environment, malnutrition, immune immaturity, and HIV-infection often synergistically affect the severity of *Cryptosporidium spp*. infection. This situation, added to socioeconomic isolation of most afflicted regions, has led to marginalization of cryptosporidiosis as a neglected disease, one that lacks an effective drug [6]. Paromomycin and nitazoxanide are considered only partially effective in otherwise healthy patients, while nitazoxanide is ineffective in AIDS patients [7].

The research efforts to find therapeutics for cryptosporidiosis are scant, relative to resources dedicated to other protozoan diseases, such as malaria. To date, only 61 structures from *Cryptosporidium spp*. (compared to almost 400 from *Plasmodium spp*.) have been deposited to the RSCB Protein Databank http://www.rscb.org. In fact, prior to our first work on *C. parvum *beginning in late 2004, only 2 *Cryptosporidium *structures had been deposited and released (both dihydrofolate reductase-thymidylate synthase). *Cryptosporidium *structure determination, is arguably a contributing step to the development of effective inhibitors and ultimately drugs. Structural genomics efforts have greatly enhanced the diversity and overall number of presently available structures by contributing over 70% of all currently available *Cryptosporidium *structures covering 34 different proteins/domains, while the remaining 30% of structures (17) only covers 5 different targets. This focus of research on a few targets, leaving many targets underexplored, plagues drug development today [8,9]. In addition, to the best of our knowledge, there have been only 4 studies to date in which a *Cryptosporidium *target and effective inhibitors have been identified and characterized. These include inosine 5'-monophosphate dehydrogenase [10], *S*-adenosylhomocysteine hydrolase [11], nonspecific polyprenyl pyrophosphate synthase (related to farnesyl pyrophosphate synthase) [12] and calcium-dependent protein kinase 1 (CDPK1) [13], where the latter two targets were contributed by structural genomics groups. In order to stimulate interest in new *Cryptosporidium *targets, we have selected for study the *C. parvum *kinome. As one of the largest protein families in eukaryotic genomes [14] and with many inhibitor libraries commercially available, protein kinases are considered attractive drug targets for human and infectious diseases alike [15]. Already, *Plasmodium *kinases are the subject of a growing body of research [16-18], as are the *Toxoplasma gondii *kinases [19]. In contrast, *Cryptosporidium parvum *PKs (*Cp*PK) are only incidentally mentioned in publications focusing on *Plasmodium *or other parasites. In an endeavour to address the void, our study spans the classification of the *C. parvum *kinome and the structural and biochemical characterization of representatives from the CDPK family and a MAP kinase. Comparison of the *Cp*PKs (protein kinases) with other known parasitic kinases illustrates some of their unique features and demonstrates that there are potential drug targets, as well as opportunities for drug design.

## Results and Discussion

### Breakdown of the *Cryptosporidium parvum *kinome

**Figure 1:**
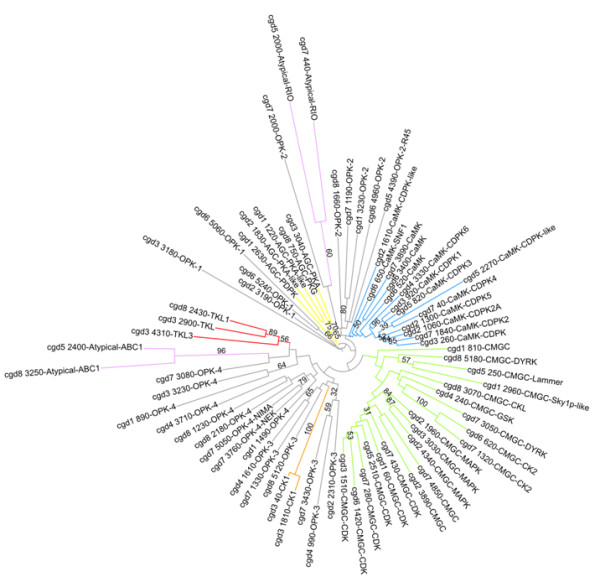
**Maximum likelihood tree from a rapid bootstrap analysis describing the classification of the 73 *C. parvum *protein kinases**. The families are colour-coded as follows: AGC (yellow), CK1 (orange), Atypical (pink), CaMK (blue), CMGC (green), TKL (red), and OPK (grey).

Assignment of the protein kinases to their subfamilies was accomplished via clustering of the kinase domain by sequence similarity. Additional information from domains outside of the catalytic domain and from evolutionary conservation was also used to aid in the analysis, culminating in a classification that rests on a hybrid of results. As such, we found 73 protein kinases with intact catalytic triads [20], including those falling into the following categories: AGC, CaMK, CK1, CMGC, TKL, Atypical, and OPK (other protein kinases). Like *P. falciparum*, there are no STE or tyrosine kinases [17], whereas only 1 STE kinase was noted in the *T. gondii *kinome analysis [19]. Of all the protein kinases found, almost a quarter (17) have no predicted orthologues outside of *Cryptosporidium spp*. The breakdown of the *C. parvum *kinome is shown in Figure 1 (and also Additional File 1, Table S1 for a summary table and Additional File 2, Table S2 for the alignments).

### AGC group

From the AGC group, 5 protein kinases were clearly identified, including the 3 cAMP-dependent protein kinases (PKA) or PKA-like kinases. The *Cp*PKA kinase cgd3_3040 is an orthologue to the *P. falciparum *and *T. gondii *PKA kinases, PFI1685w and TGME49_026030, respectively. The *Cp*PKA-like kinases include cgd1_1220 (based on its *Tg*PKA orthologue TGME49_072200) and cgd2_1830 (without predicted orthologues, but anticipated to be PKA-like from its homology with cgd1_1220). Except for *Cp*PKA and its orthologues, which share ~60% full length sequence identity, these protozoan PKA-like orthologues are quite divergent sharing less than 30% identity between them. Notably, *Cp*PKA-like kinase (cgd2_1830) is significantly shorter at the *N*-terminus and no GxGxxG motif can be identified (from subdomain I, this glycine triad structurally forms a hairpin around the bound ATP). Two of these apicomplexan PKA-like kinases (cgd1_1220 and TGME49_072200) have large *C*-terminal extensions (> 200 residues) of unknown function. Similar to *C. parvum*, *T. gondii *has a proportionally large number of PKA(-like) kinases which includes 6 of the 10 members of the AGC group [19], while 1 PKA kinase of the 5 members of the *P. falciparum *AGC group was identified [17]. Cgd7_120 is the only cAMP-dependent protein kinase regulatory subunit annotated in the *Cryptosporidium *database, CryptoDB [21], while there are 3 in *T. gondii *(TGME49_042070, TGME49_111300, and TGME49_019070) and 1 in *P. falciparum *(PFL1110c).

*C. parvum *contains a single cGMP-dependent protein kinase (*Cp*PKG), namely cgd8_750 (an orthologue to *Pf*PKG and *Tg*PKG, PF14_0346 and TGME49_111360, respectively). *Pf*PKG is essential in the blood stage and in gametogenesis of *P. falciparum *infection [22,23]. *Cp*PKG is predicted to have 3 cyclic nucleotide-binding domains (cNMP-BD) upstream of the kinase domain, while *Pf*PKG and *Tg*PKG each have 4 predicted cNMP-BDs.

The orthologue to the *T. gondii *and *P. falciparum *phosphoinositide-dependent protein kinase (PDPK) (TGME49_068210 and PF11_0227, respectively) is cgd1_2630. These are ~30% identical in sequence, but *Cp*PDPK is smaller (by ~100 residues) and without the kinase domain insert found in both the *Pf *PDPK and *Tg*PDPK. Notably, *C. parvum *does not have PKB (whereas *P. falciparum *does) or PKC (also not present in *T. gondii *or *P. falciparum*). *C. parvum *is like *P. falciparum *bearing 5 AGC protein kinases which is half of that found in *T. gondii*.

### CaMK group

**Figure 2:**
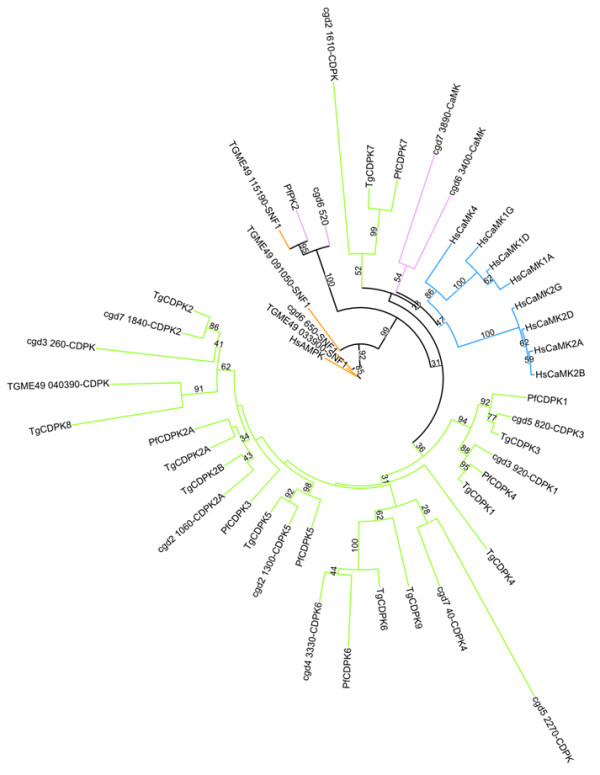
**Maximum likelihood tree from a rapid bootstrap analysis for *C. parvum *CaMK family, also including select representatives of the CaMK families from human, *P. falciparum*, and *T. gondii***. The subfamilies are as follows: CDPK (green), CaMK (blue), SNF1/AMPK (orange), and potential apicomplexan CaM-binding kinases (pink). Note that although PFB0815w is annotated as CDPK1, it is the orthologue to *Tg*CDPK3 (TGME40_105860) and PF07_0072 is annotated as *Pf*CDPK4, but is actually the orthologue to *Tg*CDPK1 (TGME49_101440).

Despite the absence of PKC, the prominence of CaMK family members indicates that regulation by calcium is clearly important in *C. parvum *parasites, as well as other apicomplexans [24]. Calmodulin (*Cp*CaM, cgd2_810) with 85% sequence identity to human CaM and 4 CaM-like proteins (containing < 220 residues and two to four EF-hands) including cgd2_3790, cgd2_1700, cgd3_3760, and cgd5_3920 were identified. From kinase domain homology, a prototypical CaMK enzyme may include cgd6_520 which was initially identified as a *Cp*CRK (CDPK-related kinase) [25]. Although it is 41% identical to and clusters with *Pf*PK2 (a proven CaMK [26]) in the phylogenetic tree (Figure 2), the auto-inhibitory helix and CaM-binding site of this *C. parvum *kinase could not be readily identified. Cgd6_3400 clusters on a sister branch to the human CaMK enzymes and is ~40% identical in sequence to them, but the auto-inhibitory helix and CaM-binding motif are not apparent in a sequence analysis. Cgd7_3890 (annotated as *Cp*CDPK7, but not containing EF-hands) contains motifs indicative of both the auto-inhibitory sequence and CaM-binding motif. Also, according to the phylogenetic tree analysis, it is related to the human CaMK enzymes.

Like in plants and ciliates, the CDPK family dominates the apicomplexan CaMK group (Figure 2). Recently the mechanism of activation of CDPKs has been elucidated by our group through structural biology using full length structures of a few apicomplexan CDPK enzymes [27,28]. Many of the *C. parvum *CDPK enzymes have already been identified including: *Cp*CDPK1 (cgd3_920), *Cp*CDPK2 (cgd7_1840), *Cp*CDPK2A (cgd2_1060), *Cp*CDPK3 (cgd5_820), *Cp*CDPK4 (cgd7_40), *Cp*CDPK5 (cgd2_1300), and *Cp*CDPK6 (cgd4_3330) [24]. A previously unidentified member of this family (cgd3_260) has a kinase domain followed by 3 predicted EF hands (see Table 1 for the domain architecture of this and all of the CaMK enzymes discussed) and clusters amongst the other CDPK enzymes of *C. parvum*. Notably, the kinase domain of cgd3_260 is > 400 residues containing a single large insert after the HRDxxxxN motif of subdomain VIB. According to OrthoMCL [29] this kinase has no known orthologues outside of *Cryptosporidium spp*. Also among the CDPK enzymes is cgd5_2270 (annotated as MELK (maternal embryonic leucine zipper kinase)), clustering with *Cp*CDPK4, but without EF-hands and with small *N*- and *C*- terminal extensions of ~25 residues each. Clustered with the CDPK7 enzymes is cgd2_1610, which also does not have any EF-hands. Cgd6_650 is the *Cp*SNF1 (homologous to the AMP-modulated protein kinases (AMPK)) according to its clustering on the CaMK phylogenetic tree presented herein (Figure 2) and its *Tg*SNF1 orthologue (TGME49_033900). Upon comparison to *P. falciparum *with 13 CaMKs [17], there are 15 for *C. parvum *including the 8 CDPK enzymes with intact EF-hands, while *T. gondii *has 20 CaMKs listed with complete catalytic triads and 16 categorized as CDPKs [19].

**Table 1:** Domain architecture of CaMK family members from *C.parvum*, *T. gondii*, and *P. falciparum*.

Annotation	ID	Architecture	KD size	FL size
*Cp*CaMK	cgd6_520	----KD-----	241	1103
*Pf*PK2	PFL1885 c	-KD-	253	509
*Tg*SNF1	TGME49_115190	--KD---	253	774
*Cp*CaMK	cgd6_3400	KD----------	279	979
*Cp*CaMK	cgd7_3890	KD	267	357
*Cp*CDPK-like	cgd2_1610	KD	340	516
*Cp*CDPK-like	cgd5_2270	-KD	278	327
*Cp*CDPK	cgd3_260	KD----EF.EF	426	1180
*Tg*CDPK	TGME49_040390	-----KD-----------EF.EF	562	2483
*Cp*CDPK1	cgd3_920	KD.EF.EF.EF.EF	256	538
*Pf*CDPK1	PFB0815w	KD.EF.EF.EF.EF	269	524
*Tg*CDPK1	TGME49_101440	KD.EF.EF.EF.EF	332	582
*Cp*CDPK2	cgd7_1840	CMB20.KD.EF.EF.EF.EF	254	676
*Tg*CDPK2	TGME49_025490	CMB20.KD.EF.EF.EF.EF	254	711
*Cp*CDPK2A	cgd2_1060	--KD.EF.EF.EF.EF	255	718
*Tg*CDPK2A	TGME49_006590	--KD.EF.EF.EF.EF.EF	254	761
*Pf*CDPK2B	PFF0520w	KD.EF.EF.EF	254	509
*Tg*CDPK2B	TGME49_042400	-KD.EF.EF.EF.EF	254	604
*Cp*CDPK3	cgd5_820	KD.EF.EF.EF.EF	256	523
*Pf*CDPK3	PFC0420w	-KD.EF.EF.EF.EF	255	562
*Tg*CDPK3	TGME49_105860	KD.EF.EF.EF.EF	257	537
*Pf*CDPK4	PF07_0072	KD.EF.EF.EF.EF	258	528
*Tg*CDPK4	TGME49_037890	------KD.EF.EF.EF	350	1171
*Cp*CDPK4	cgd7_40	-KD(ZnF).EF.EF.EF--	306	824
*Tg*CDPK4A	TGME49_095760	KD.EF.EF.EF	266	534
*Cp*CDPK5	cgd2_1300	--KD.EF.EF.EF.EF	254	677
*Pf*CDPK5	PF13_0211	-KD.EF.EF.EF.EF	254	568
*Tg*CDPK5	TGME49_024950	--KD.EF.EF.EF.EF	254	681
*Cp*CDPK6	cgd4_3330	EF.EF.KD.EF.EF.EF	259	622
*Pf*CDPK6	PF11_0239	-----------EF.EF.KD.EF.EF.EF	252	1617
*Tg*CDPK6	TGME49_018720	EF.EF.KD.EF.EF.EF.EF	258	557
*Pf*CDPK7	PF11_0242	EF.EF----------------PH.KD--	256	2265
*Tg*CDPK7	TGME49_028750	------PH--KD----	257	1346
*Tg*CDPK8	TGME49_092060	-KD	457	624
*Tg*CDPK9	TGME49_017600	-EF.KD	298	488
*Cp*SNF1	cgd6_650	KD------	252	859
*Tg*SNF1	TGME49_033900	KD-	252	412
*Tg*SNF1	TGME49_091050	-----KD-	252	827

Each "-" within the architecture column represents up to 100 residues. Abbreviations include: FL for full length, KD for kinase domain, EF for EF-hands, ZnF for zinc finger, CMB20 for carbohydrate-binding module, and PD for *Pleckstrin *homology domain.

### CK1 group

Parasitic CK1 enzymes, including those from *P. falciparum *and *T. gondii *have attracted attention because of their unexpected binding to an immobilized cyclin-dependent protein kinase (CDK) inhibitor (purvalanol B) [30]. Two *Cp*CK1 enzymes have been identified herein, in comparison to 1 and 3 from *P. falciparum *and *T. gondii*, respectively [17,19]. Specifically, from *T. gondii *the cytosolic *Tg*CK1α and the membrane bound *Tg*CK1β were isolated, while the third *Tg*CK1 (TGME49_047710) is uncharacterized. *Tg*CK1α could be selectively inhibited by purvalanol B and aminopurvalanol A over the host CK1 enzymes; and importantly, inhibition by aminopurvalanol (the more cell-permeable of the two) inhibits parasite cell growth [31]. All of these parasitic CK1 kinases share high sequence identity in the kinase domain (64% to 89%) and should be tested for similar inhibition profiles, including CDK inhibitors. Similar to *Tg*CK1β which has a *C*-terminal tail implicated in membrane localization [31], both *C. parvum *enzymes have *C*-terminal tails indicating potential membrane localization.

### CMGC group

There are 20 CMGC kinases in *C. parvum *which include the cyclin-dependent- (CDK), mitogen-activated- (MAPK), glycogen-synthase- (GSK), and CDK-like (CDKL) kinases, as well as CK2 (casein kinase 2), CLK (CDC-like kinase), DYRK (dual-specificity tyrosine-(Y)-phosphorylation regulated kinase), and SRPK (serine/arginine-rich protein-specific kinase) [32]. In comparison, there are 20 CMGC kinases from *T. gondii *and 18 from *P. falciparum *[17,19]. Like those studied from *P. falciparum *and in other eukaryotic systems, a majority of CMGC kinases are involved in the control of cell proliferation and development, so their relative abundance in these organisms may reflect the variety of successive proliferative and non-proliferative stages which constitute their life cycles. CDK enzymes with the typical PSTAIRE cyclin-binding motif (where the terminal glutamate is the conserved residue in subdomain III forming a salt bridge with the catalyic lysine of subdomain II) include cgd3_1510 (which has no known orthologues outside *Cryptosporidium spp*.) and cgd5_2510 (orthologue of *Pf*PK5 (MAL13P1.279) and *Tg*PK2 (TGME49_018220)). Two other CDK enzymes were identified through their orthologues including cgd6_1420 with a SKTAIRE motif (orthologue to PFD0740w, CDC2-related protein kinase 3) and cgd7_430 with a HFTVLRE motif (orthologue to PF10_0141, MO15-related protein kinase (*Pf*MRK) and TGME49_070330, CaMK). Note that "composite" kinases (*i.e. *those having characteristics from two different groups) have been previously characterized in apicomplexan parasites [33]. As well, there are cgd7_280 and cgd1_60 which have no known orthologues outside of *Cryptosporidium spp*., but are annotated as CDKs in CryptoDB, presumably due to the presence of PATSIRE and STTTLRE motifs. With respect to the first of 3 of the these *Cp*CDK enzymes with apicomplexan orthologues, the putative *Cp*MRK (cgd7_430) enzyme and its orthologue, *Pf*MRK (which has sequence homology to human CDK) are the same size (320 and 324 residues, respectively) and share 50% sequence identity indicating that classes of compounds effective against *Pf*MRK (of which there are several) should also be tried against *Cp*MRK. These include the purines, quinolinones, oxindoles, and chalcones which have sub-micromolar IC_50 _values against the *Plasmodium *enzyme, but not the human CDK enzymes tested [34]. Also, *Pf*MRK is inhibited by bromohydrosulfonylacetamides which possess moderate antimalarial activity against drug resistant parasites, but not broad spectrum CDK inhibitors [35]. *Pf*MRK and its effector *Pf*MAT1 (where the *C*-terminal domain of *Pf*MAT1 is the *Pf*MRK activator domain) have been demonstrated to be co-localized to the parasite nucleus and that *Pf*MRK phosphorylates two plasmodial DNA replication proteins suggesting that *Pf*MRK in the nucleus is involved with the regulation of the DNA replication machinery [36]. CDK enzyme, cgd6_1420 is half the size of its orthologue, *Pf*CRK-3 (PFD0740w) with 1339 residues, which has been implicated as crucial in intraerythrocytic development of *P. falciparum *via regulation of gene expression [37]. Although *Pf*CRK-3 bears an *N*-terminal domain of between 350-400 residues (depending on how the > 10 inserts are counted), these enzymes still share 36% sequence identity. *Cp*CDK (cgd5_2510) and its orthologues are approximately the same size (at 295, 288, and 300 residues for *C. parvum*, *P. falciparum*, and *T. gondii*, respectively) and share from 65% to 72% sequence identity. Crystal structures of *C. parvum *CDK (cgd5_2510) have been solved with indirubin 3'-monoxime [PDB: 2QKR] and ADP [PDB: 3NIZ] bound by our group, as well as its orthologue structure (*Pf*PK5) structure in the presence [PDB: 1V0P and 1V0O] and absence of inhibitors [PDB: 1OB3] by others [38].

*C. parvum *has 3 MAP kinases including cgd2_1960 (which is the orthologue of PF14_0294 (*Pf*MAP-1)), cgd2_4340 (orthologue of PF11_0147 (*Pf*MAP-2) and TGME49_007820), and cgd3_3030 (orthologue to TGME49_112570, *Tg*MAPK-1). Each of the MAP-2 orthologues is similar in size from 508 to 563 residues, sharing from 49% to 55% sequence identity. Interestingly, like *Pf*MAP-2, the *C. parvum *and *T. gondii *MAP-2 orthologues share the peculiarity of not possessing the conserved TXY activation motif (just upstream of the conserved APE motif of subdomain VII) usually found in enzymes of this family [39]. Instead, each has a TSH at the same location; and furthermore no vertebrate MAPK enzymes deviate in this activation motif suggesting that the fine regulation mechanisms of these apicomplexan MAP-2 orthologues may present a collective opportunity for drug targeting. The remaining two *C. parvum *MAP kinases have the expected TxY motif, both in the form of TDY, like their respective *P. falciparum *and *T. gondii *orthologues. As well, using a reverse genetics approach, *Pf*MAP-2 gene was shown to be essential for completion of the asexual cycle of *P. falciparum*, an unexpected result in view of the non-essentiality of the orthologous gene for *P. berghei *erythrocytic schizogony [40]. The *Pf*MAP-1 and putative *Cp*MAP-1 orthologues only share 38% identity, but both have substantial *C*-terminal extensions of 602 and 338 residues, respectively. The crystal structure of the kinase domain of putative *Cp*MAP-1 [PDB: 3OZ6] has been solved by our group and is discussed below. Although cdg3_3030 and *Tg*MAP-2 share 42% sequence identity, they are notably distinct in size (overall, 566 versus 1212 residues, respectively) where the difference in size can be mostly attributed to the uncharacterized large *C*-terminal extensions of 247 and 794 residues, respectively.

Both the *Pf*GSK-3 and *Cp*GSK-3 (cgd4_240) bear an unusual *N*-terminal extension of about 70 residues (although there is no sequence conservation between the extensions and the catalytic domains are well-conserved). Notably, *Cp*GSK-3 has an insert between the catalytic lysine and just upstream of the gatekeeper motif. Its structure has been solved [PDB: 3EBO] by our group. Although the physiological functions of *Pf*GSK-3 remain to be elucidated, a series of GSK-3β inhibitors tested on both *Pf*GSK-3 and mammalian GSK-3β show a partially divergent sensitivity [41]. These results give promise to both *Pf*GSK-3 and *Cp*GSK-3 with respect to drug discovery.

Ten other members of the CMGC group were identified, including cgd8_3070 from its orthologue (TGME49_085160, a putative cyclin-dependent kinase-like 5). The putative *Cp*CKL and *Tg*CKL-5 share 41% sequence identity; however, the *C. parvum *enzyme is significantly larger (551 residues versus 351 residues for *Tg*CKL-5) with > 10 inserts relative to its *T. gondii *orthologue. In addition, a LAMMER kinase, 2 DYRK kinases, and a Sky1p kinase were identified. Characterization of the *Pf*LAMMER describes the enzyme as comprised of 2 domains, where the *N*-terminal domain is unique and containing multiple consensus phosphorylation sites, a number of RS/SR dipeptides, a large portion of charged residues, two putative nuclear localization signals, and 14 copies of a DKYD repeat and the *C*-terminal domain is typical of the LAMMER family [42]. By comparison, *Cp*LAMMER (cgd5_250) has a smaller *N*-terminal domain comprised of ~300 residues (versus 550 for the *Pf*LAMMER and ~1330 for *Tg*LAMMER), has a HTD motif (subdomain X), and is unusually rich in asparagine residues. The *Pf*LAMMER is expressed specifically in the sexual stage; and thus the authors concluded that it might be important in the regulation of sexual differentiation [42]. *C. parvum *CMGC kinases belonging to DYRK subfamily include: cgd7_3050 bearing an HCD motif (orthologue of *Tg*DYRK, TGME49_004280) and cgd8_5180 bearing a HAD motif (orthologue of *Pf*DYRK, PF11_0156 and of another *Tg*DYRK, TGME49_113180). These apicomplexan DYRK enzymes have low sequence identity between them and variable *N*-terminal domains ranging in size from almost 150 residues to over 700 residues. Cgd1_2960 is annotated as Sky1p-like and is implicated in RNA metabolism. The arginine of the HRD motif (subdomain X) is not conserved and is replaced by threonine. Although it has a small *N*- and *C*-terminal tails of 81 and 65 residues, respectively, it is the 4 inserts within the kinase domain that make this enzyme stand out, including one of almost 250 residues just upstream of the DFG motif of kinase subdomain VII. CK2 enzymes are the only family within the CMGC group that replaces the CMGC-arginine (found just upstream of the APE motif of subdomain VIII) with a lysine, as is observed herein for the *C. parvum *enzymes and their orthologues, cgd6_620 (orthologue of PF11_0096 CK2α and TGME49_063070, CK2) and cgd7_1320, which has no known orthologues outside of *Cryptosporidium spp*. This CK2 specific motif may allow for the phosphorylation of substrates with proline or nonproline residues at the P + 1 position of the substrate due to its increased side-chain flexibility, giving H-bonding to the main-chain oxygen at the strained position that the CMGC-arginine does, but also accommodating alterative H-bonding [43]. The conserved CK2 glycine following it may further contribute to the flexibility allowing a greater range of main-chain conformations, although cgd7_1320 does not conserve this glycine. The *Pf*CK2α has been characterized with protein kinase activity; and gene disruption experiments have shown that it is crucial to the asexual blood stage in *Plasmodium*, thereby providing evidence that it may be a drug target [44]. Furthermore, it has differential susceptibility to a small molecular inhibitor making it an attractive target for antimalarial intervention, and potentially its orthologues in other parasitic diseases. Finally, 3 uncharacterized CMGC members with no known orthologues outside of *Cryptosporidium spp*. were also identified: cgd1_810 (which has a > 600 residue *N*-terminal extension and > 600 residue kinase domain), cgd7_4850 (which is relatively small at 286 residues, has an asparagine instead of an arginine in the HRD motif), and cgd2_3890.

### Atypical group

Atypical protein kinases (aPKs) lack sequence similarity to the eukaryotic protein kinase domain hidden Markov model profile and as such are unrelated (or only distantly related) by sequence to ePKs; however, they have been shown experimentally to have protein kinase activity or are clear homologues of aPKs with demonstrated protein kinase activity. There are four *C. parvum *kinases that are identified as atypical based on their *P. falciparum *and *T. gondii *orthologues, including 2 RIOs (cgd7_440 and cgd5_2000) and 2 ABC1s (cgd8_3250 and cgd5_2400) (Additional File 1, Table S1). There may be as many as 24 from *T. gondii *[19] and 4 from *P. falciparum *[17] (including the RIOs and ABC1s).

### TKL group

In *C. parvum*, there are 3 TKL enzymes including cgd8_2430, cgd3_2900, and cgd3_4310 with each having *P. falciparum *and/or *T. gondii *TKL orthologues, while *T. gondii *and *P. falciparum *each contains 5 TKL enzymes (Additional File 1, Table S1). Recently, work on *Pf*TKL-3 (PF13_0258, the orthologue of cgd3_4310 (the putative *Cp*TKL-3) has demonstrated that it is essential for asexual parasite proliferation in human erythrocytes [45]. Furthermore, the authors showed that it undergoes *in vitro *autophosphorylation and phosphorylates exogenous substrates both of which are dependent on the presence of a sterile α-motif (SAM) domain at the *N*-terminus. Although these *C. parvum *and *P. falciparum *TKL-3 orthologues only share 30% overall sequence identity, they both have a SAM domain, as well as putative MORN (membrane occupation and recognition nexus) motifs that are *N*-terminal to the kinase domain and not shared by the *Tg*TKL-3 orthologue. In the case of the *P. falciparum *orthologue, there is an additional *N*-terminal domain of > 1300 residues (bearing PEXEL and 14_3_3 mode II binding motifs) upstream of the SAM domain, correspondingly this domain is only ~300 residues in the *C. parvum *enzyme. Although *Cp*TKL-1 and *Pf*TKL-1 (cgd8_2430 and PFB0520w, respectively) also bear these SAM and MORN motifs, the third *Cp*TKL does not (cgd3_4310).

### OPK group

There are 2 clades of protein kinases entirely unique to *Apicomplexa*, namely rhoptry kinases (*T*. *gondii *and *Neospora caninum *only) and FIKK kinases (found only in *Plasmodium*). We conducted BLAST analysis of the sequences of all known *Toxoplasma *ROP kinases against the *C. parvum *genome and did not find a single orthologue. However, amongst a clad of others, a FIKK- containing kinase was identified (cgd5_4390) which has been previously annotated as a R45-like orthologue (and named *Cp*R45) by Schneider *et al. *from a tBLASTn search using the protein kinase domain of PFD1175w [46]. This protein is phylogenetically close to the *Plasmodium *FIKK cluster and also 40% identical in sequence to *Pf*FIKK8 (MAL8P1.203), with both lacking the PEXEL motif found in most other *P. falciparum *FIKK kinases. It is also the orthologue of a catalytically incomplete *Tg*FIKK kinase (TGME49_089050). Although there are clades of OPK enzymes amongst the annotated protein kinases of *C. parvum*, unique conserved motifs potentially identifying a unique *Cryptosporidium *class of protein kinases are not readily apparent. Since *Cp*OPK enzymes comprise almost 35% of the kinome, we have divided them into their respective clades to simplify the discussion and are not implying a further organization of the *C. parvum *kinome at this point. OPK clade 1 is comprised of 4 protein kinases including: cgd2_3190 (with a > 300 residue *N*-terminal extension) which is the orthologue of a putatively identified AGC-related PFC0385 c from *P. falciparum *and the *T. gondii *aurora kinase TGME49_003010; cgd6_5060 which contains *N*- and *C*-terminal extensions of ~200 residues and has a *P. falciparum *orthologue that is also AGC-related; cgd6_5240 which has no known orthologues outside of *Cryptosporidium spp*.; and cgd3_3180. OPK clade 2 includes 6 enzymes: cgd7_2000 which has no known orthologues outside of *Cryptosporidium spp*. and *N*- and *C*-terminal extensions of 436 and 72 residues; *Cp*R45 (already discussed); cgd7_1190 which is an orthologue of PFC0485w (CaMK) and TGME49_018550 (PIK3R4 kinase-related protein); cgd1_3230 which has no known orthologues and a *C*-terminal extension that is > 500 residues; cgd6_4960; and cgd8_1660. OPK clade 3 contains 6 kinases that very little is known about including: cgd4_990 and cgd4_1610, both with no known orthologues outside of *Cryptosporidium spp*; cgd8_5120 which may be related to the CMGC kinases based on the annotations of its orthologues; cgd7_1330 which may contain an *N*-terminal transmembrane domain and has green plant/algae orthologues; cgd7_3430 which has a TKL orthologue from *T. gondii*, and cgd2_2310 with uncharacterized apicomplexan orthologues. OPK clade 4 contains cgd1_1490 (with a > 250 residue *C*-terminal domain) which is an orthologue to PFL1370w (NIMA related protein kinase 1) that also has a *C*-terminal extension (> 750 residues). *Pf*NEK-1 is able to autophosphorylate and phosphorylate select protein substrates, including *Pf*MAP-2 [47]. There is also cgd1_890 which has a kinase domain with a large insert making it more than twice the size (at 659 residues) of a typical kinase domain. It is the orthologue to PFF1370w (*Pf*PK4) and TGME49_029630 (kinase with an incomplete catalytic triad). Cgd3_3230 has substantial *N*- and *C*-terminal extensions (284 and 920 residues, respectively) and is the orthologue to PF14_0423 (eukaryotic initiation factor 2 alpha kinase 1). Cgd7_3760 is an orthologue to NEK kinases from *P. falciparum *and *T. gondii*. Cgd7_5050 is annotated as NIMA related kinase 5 and contains an *N*-terminal domain > 1000 residues. Interestingly, cgd4_3710 has an unusually large (> 400 residues) kinase domain (typically ~255 residues) that is a function of 4 inserts including one after the catalytic lysine (from subdomain II), another after the HRDxxxxN motif of subdomain VIB, one after the APE motif of subdomain VIII, and the last after the conserved aspartic acid of subdomain IX. The remaining 3 kinases include cgd7_3080 which has a *T. gondii *Wee kinase orthologue, cgd8_1230, and cgd8_2180.

### Activity of *Cp*CDPK1, *Cp*CDPK2, *Cp*CDPK3, and *Cp*CDPK4

The effect of calcium on the activity of constructs containing the kinase domain (KD) and CAD (CDPK activation domain) of *Cp*CDPK1, *Cp*CDPK2, *Cp*CDPK3, and *Cp*CDPK4 was tested using the pyruvate kinase-lactate dehydrogenase (PK-LDH) assay and the peptide substrate Syntide-2 (a common CaMK substrate with the sequence PLARTLSVAGLPGKK). They exhibited a range of phosphorylation activities (Table 2), but all showed an increase in activity corresponding to an increase in calcium concentrations with turnover numbers of 11 ± 1, 9 ± 2, 64 ± 6, and 3 ± 1 mM^-1^min^-1^, respectively, and in the range of values previously determined for other CDPKs with the exception of *Cp*CDPK3 [28]. The difference in the catalytic efficiencies of these four CDPKs is on the order of 300-fold for the same common kinase peptide substrate, Syntide-2. Indeed, the CDPK enzymes are expected to have different substrate specificities. For example *Pf*CDPK1 is capable of phosphorylating myelin basic protein (MBP), histone 1, and casein, while *Pf*CDPK2 only recognizes MBP as a substrate [48].

**Table 2:** Kinetics of *Cp*CDPK1 (cgd3_920, true FL version), *Cp*CDPK2 (cgd7_1840), *Cp*CDPK3 (cgd5_820), and *Cp*CDPK4 (cgd7_40).

Enzyme	K_M _syntide (μM)	K_M _ATP (μM)	k_cat_ (min^-1^)	k_cat_/K_M_ (mM^-1^.min^-1^)
*Cp*CDPK1	157 ± 17	20 ± 3	1370 ± 40	8726
*Cp*CDPK2	260 ± 13	22 ± 1	240 ± 5	923
*Cp*CDPK3	156 ± 69	81 ± 4	367 ± 37	2353
*Cp*CDPK4	426 ± 47	29 ± 8	12 ± 1	28

### Effect of the *N*-terminal latch on CDPK activity

The *Cp*CDPK1 construct tested herein has a complete *N*-terminal domain comprising 55 additional residues over the other three CDPK enzymes tested, where these constructs expressed do not have this *N*-terminal domain. The CDPK *N*-terminal domain has been postulated to function as a structural latch that will enable full kinase activity to be maintained once calcium has been depleted. Our data on *Cp*CDPK1 in the presence of calcium shows that there is little difference between the activity of a construct with an intact latch versus a construct without a latch, a difference of ~50 residues upstream of the subdomain 1 GxGxxG motif (data not shown). However there is some sequence conservation of hydrophobic residues just upstream of the GxGxxG motif for *P. falciparum*, *T. gondii*, and *C. parvum*; and this may indicate a conserved latch regulatory mechanism for apicomplexan CDPKs [28]. Specifically, hydrophobic patterns including a PGMF motif in at least 6 apicomplexan CDPKs and a FxRxxFILxxxG (or the variation of FxRxxooxxxxL), where x is any residue and o is a hydrophobic residue, in 17 CDPKs which may signify that the application of such a regulatory mechanism reliant on the interaction of hydrophobic residues is used by apicomplexan CDPKs (Additional File 2, Table S4).

### Inhibition of *Cp*CDPK1 by pyrazolopyrimidine derivatives

Inhibition of *Cp*CDPK1 was thoroughly investigated by screening with a series of compounds designed to exploit the small gatekeeper that is naturally occurring in *Cp*CDPK1, but not in the other *Cp*CDPKs (Additional File 3, Table S5). For example, similar to previous results [13], pyrazolopyrimidine derivatives are ineffective against kinases with bulky gatekeepers (found just downstream of the catalytic lysine in a Yoo*xooxGGELFxxI motif, where o is hydrophobic, x is any, and * is the gatekeeper), but *Cp*CDPK1 is expected to be sensitive to such inhibitors owing to the presence of a glycine instead of the typical methionine present in the remaining *Cp*CDPKs. Accordingly, over a dozen PP1 derivatives were found that inhibit *Cp*CDPK1 with IC_50_'s less than 10 nM (Table 3).

**Table 3:** IC_50 _values for *Cp*CDPK1 inhibitors tested.

Compound	IC_50 _(nM)
	1.3 ± 0.2
	2.1 ± 0.2
	2.1 ± 0.3
	2.8 ± 0.2
	3.4 ± 0.1
	3.4 ± 0.1
	0.9 ± 0.1
	2.6 ± 0.1
	4.7 ± 0.4
	7 ± 0.2
	5.6 ± 0.5
	15.2 ± 4.4
	5.6 ± 0.8
	7.7 ± 1
	22 ± 2
	249 ± 73
	96 ± 13
	88 ± 0.14
	616 ± 203
	2722 ± 72

All compounds were tested using 500 μM ATP and 500 μM Syntide2 as described in Methods. A concentration range from 1 nM to 1 μM for all compounds was used except for CZ25, CZ15 and 1nA-PP1 (2 nM to 2 μM), CZ27 (4 nM to 4 μM) and JC022 (8 nM to 8 μM).

### Structural characterization of select *Cp*CDPKs

**Figure 3:**
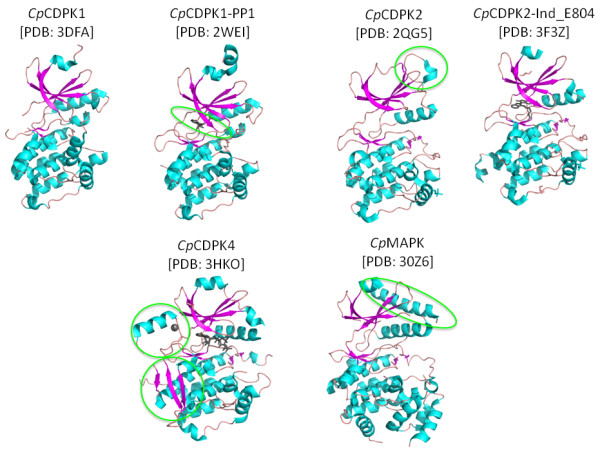
**Overall structures of *Cp*CDPK enzymes**. The green circles highlight important features within the structures. In the *Cp*CDPK1 structure, the binding of the PP1 inhibitor is shown. The α-helix D orientation is highlighted in the apo *Cp*CDPK2 structure. For the *Cp*CDPK4 structure, the Zn-finger and β-mesh position are shown. In the *Cp*MAP-1 structure, the unusually long α-L16 helix is shown.

The CDPKs have a range of different type of domain organizations, as shown in Table 1. To date, *C. parvum *and *T. gondii *CDPK structures solved include KD, CAD (including 4-EF hands), and intact KD-CAD (with and without calcium bound). Herein, we present the solved KD structures from *Cp*CDPK1, *Cp*CDPK2, and *Cp*CDPK4 (Figure 3 and Table 4).

**Table 4:** Data collection, phasing, and refinement statistics for the *C. parvum *CDPK and MAP-1 structures.

Structure		*Cp*CDPK1	*Cp*CDPK1-PP1	*Cp*CDPK2
PDB Code		3DFA	2WEI	2QG5

Space Group		P43212	P43212	C2

Cell Dimensions				

	a (Å)	69.45	68.9	138.18

	b (Å)	69.45	68.9	86.48

	c (Å)	137.41	130.46	87.96

	Α (°)	90	90	90

	β (°)	90	90	96.37

	γ (°)	90	90	90

Wavelength		0.91996	0.99989	1

Resolution (Å)		50 - 2.45 (2.54)	33 - 1.65 (1.74)	50 - 2.3 (2.36)

Unique reflections			38607	38421

R_merge_		.072 (.471)	.11 (.70)	.11 (.45)

I/σI		11.2 (4.53)	10.2 (2.3)	12.4 (1.5)

Completeness (%)		96.9 (83.5)	99.9 (100)	88.4 (60.7)

Redundancy		13.2 (11.1)	8.3 (7)	3.4 (2.0)

**Refinement**				

Resolution		50 - 2.45 (2.52)	33 - 1.65 (1.693)	40 - 2.3 (2.36)

Number of Reflections		12590	36676	38421

Test Set		611	1837	2104

R_work_/R_free_		.234/.263	.197/.229	.224/.286

Number of Atoms		2183	2486	7227

Mean B_factor_		59.4	22.1	38.03

Ramachandran Favored		96.1	97.5	95.8

Ramachandran Disallowed		0	0	0

RMS deviations				

	Bond length (Å)	0.007	0.014	0.013

	Bond angle (°)	1.014	1.41	1.351

**Structure**		*Cp*CDPK2-Ind-E804	*Cp*CDPK4	*Cp*MAP-1

PDB Code		3F3Z	3HKO	3OZ6

Space Group		C2	P212121	P212121

Cell Dimensions				

	a (Å)	63.32	54.48	74.30

	b (Å)	82.86	63.04	96.75

	c (Å)	62.15	84.04	119.99

	Α (°)	90	90	90

	β (°)	111.61	90	90

	γ (°)	90	90	90

Wavelength		1.5418	0.978	0.97924

Resolution (Å)		50 - 1.84 (1.91)	50 - 1.80 (1.86)	50 - 2.4 (2.44)

Measured reflections				607387

Unique reflections			27359	62534

R_merge_		0.054 (.606)	0.073 (.555)	0.087 (.921)

I/σI		X (2.5)	20.5 (1.53)	14.8 (1.34)

Completeness (%)		99.2 (95.7)	99.2 (92.4)	99.2 (98.7)

Redundancy		3.7 (3.5)	6.1 (2.50)	3.8 (3.8)

**Refinement**				

Resolution		50 - 1.84 (1.89)	50 - 1.8 (1.84)	45 - 2.4 (2.44)

Number of Reflections		25653	27292	33427

Test Set		1303	1376	1697

R_work_/R_free_		.189/.230	.204/.246	.232/.259

Number of Atoms		2462	2789	5390

Mean B_factor_		27.47	26.95	51.1

Ramachandran Favored		98.2	97.2	96.7

Ramachandran Disallowed		0	0	0

RMS deviations				

	Bond length (Å)	0.01	0.008	0.01

	Bond angle (°)	1.501	1.204	1.100

### *Cp*CDPK1 and *Cp*CDPK3 structures

**Figure 4:**
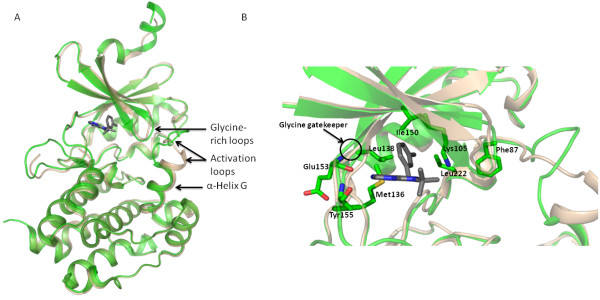
**Comparison of *Cp*CDPK1 structures**. (A) Overall comparison of *Cp*CDPK1 in apo form [PDB: 3DFA] (beige) and *Cp*CDPK1- PP1-derivative bound [PDB: 2WEI] (green). (B) Active sites of *Cp*CDPK1 structures showing some of the residues (numbering from the PP1-bound structure PDB: 2WEI) involved in binding a PP1-derivative.

The structures of *Cp*CDPK1 [PDB: 3IGO] and *Cp*CDPK3 [PDB: 3LIJ] with both the KD and CAD domains intact have been solved by our group. These and other CDPK structures including calcium-free and calcium-bound forms have been used to describe a model for the activation of the CDPK family of enzymes and to characterize the CAD domain, a novel member of the EF-hand containing family, whose structure has also been solved for *Cp*CDPK3 [PDB: 3L19] [27,28]. The exploitation of the *Cp*CDPK1 ATP-binding site featuring a glycine-gatekeeper has also been described in detail including its corresponding full length kinase structure with inhibitors bound [PDB: 3NCG and 3MWU] [13]. Herein, we have solved the KD structure of *Cp*CDPK1 [PDB: 3DFA] in apo form as well as with a PP1-derivative (1-*tert*-butyl-3-(3-methylbenzyl)-1H-pyrazolo[3,4-d]pyrimidin-4-amine, 3MB-PP1) bound [PDB: 2WEI]. The overall fold of the KD structures with and without the PP1 derivative bound are similar (Figure 4A). In both structures, the glycine-rich loop is clamped down reflective of the activated form, the tip of which (containing Phe87, all numbering from full length and same as for PDB: 2WEI) is tucked into a pocket created by the crossing of β-sheet 3 and the residues just prior to the activation loop. The activation loop, which moves closer to the active site in the 3MB-PP1 structure sits lower and retains an additional turn at the top of the α-helix G in the apo structure. The pyrazolo-pyrimidine in the PP1 molecule occupies the same space where the adenine in ATP is typically found, forming H-bonds with the backbone of Glu153 and Tyr155 (Figure 4B). N8 of the 3MB-PP1 is linked by a water to the activating Lys105. The large, hydrophobic methylbenzyl group of 3MB-PP1 sits deep within the pocket lined by Leu222, Leu138, Ile150, and Met136. In most other kinases, this pocket is ablated by the side-chain of a large gatekeeper residue.

### *Cp*CDPK2 structure

**Figure 5:**
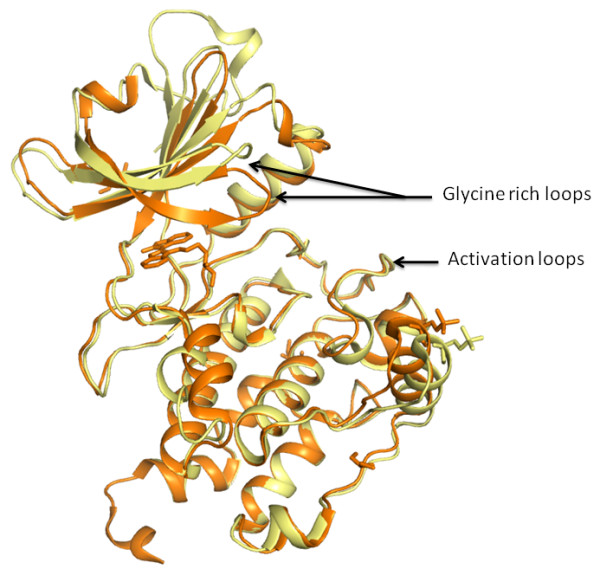
**Comparison of *Cp*CDPK2 structures**. *Cp*CDPK2 in apo form [PDB: 2QG5] (yellow) and *Cp*CDPK2- indirubin E804 bound [PDB: 3F3Z] (orange) shows the shift in the glycine-rich loop.

*Cp*CDPK2 comprises an *N*-terminal domain that is predicted to bind a carbohydrate, KD, and the CAD. We have solved the crystal structures of *Cp*CDPK2 KD with indirubin E804 (3-({[(3*S*)-3,4-dihydroxybutyl]oxy}amino)-1H,2'H-2,3'-biindol-2'-one) bound [PDB: 3F3Z], as well as the apo form [PDB: 2QG5]. Both structures have completely ordered activation loops, with α-helix C of the indirubin-bound structure not completely in the activated form (Figure 5). There is a dramatic difference in glycine-rich loops between the apo and the indirubin-bound structures. In the apo form, the loop is moved up and away from the activation site, adopting a conformation less amenable to binding ATP. With the indirubin-bound structure, the backbone atoms from the loop move up to 8 Å closer to the active site. Once again, the indole moiety is interacting with the backbone hinge residues Glu103 and Cys105 (numbering is identical in both structures, but 194 less than full length numbering). The hydroxyl groups from the "tail" of the indirubin form a series of H-bonds with residues from the *C*-lobe of the catalytic domain. Glu109 is pulled up by this interaction, such that the α-helix D is more ordered and contains an additional turn compared to the α-helix D from the apo structure.

### *Cp*CDPK4 structure

**Figure 6:**
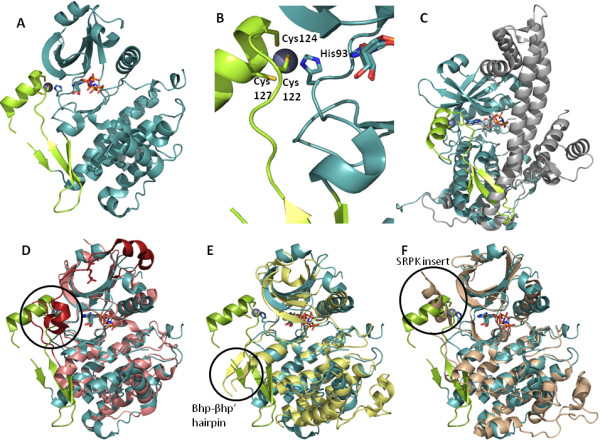
**Structural analysis of *Cp*CDPK4**. (A) Overall view of *Cp*CDPK4 [PDB: 3HKO] with the zinc finger and β-mesh insert highlighted in lime. (B) Zn-finger of *Cp*CDPK4 is shown with the residues involved in zinc binding noted. (C) Overlay of aligned views of *Cp*CDPK4 with the *C*-terminus of the KD and entire CAD of *Tg*CDPK1 (for visual clarity the *N*-terminal portion of the KD is not shown) in grey [PDB: 3KU2]. (D) An overall structural comparison of *Cp*CDPK4 (teal/lime) and PKC-ι (salmon/magenta) [PDB:3AX8] with the *C*-terminal tail (from K531) highlighted in magenta and a circle indicating its overlap with *Cp*CDPK4. (E) An overall structural comparison of *Cp*CDPK4 (teal/lime) and human CLK1 (pale yellow) [PDB:1Z57]. (F) An overall structural comparison of *Cp*CDPK4 (teal/lime) and human SRPK (beige) [PDB:1WBP].

The KD structure of *Cp*CDPK4 has been solved in the presence of a non-hydrolyzable ATP analogue (phosphoaminophosphonic acid-adenylate ester) [PDB: 3HKO]. Sequence alignment shows that *Cp*CDPK4 has a unique insert within the kinase domain that is particularly cysteine-rich in the centre: ^101^LNVFIDDSTGKCAMDVVKTQICPCPECNEEAINGSIHGFRES^140 ^(with all numbering from PDB: 3HKO which is 125 less than full length numbering). This insert is situated between the hinge region and α-helix F. It consists of an anti-parallel β-mesh that interacts with the helical *C*-lobe of the KD and a helix that interacts with the *N*-terminal β-lobe (Figure 6A). Embedded in the helix is a zinc ion coordinated by His93, Cys122, Cys124 and Cys127 (Figure 6B). This zinc finger is not present in any other known protein KD, based on searches by sequence and structure (also from personal communications with Gerald Manning and Stefan Knapp), making the insert particularly interesting and unique.

When *Cp*CDPK4 is overlaid with the structure of PKC-ι [PDB: 3A8W] that includes the *C*-terminal residues, we see that the zinc finger lies within the same vicinity as the *C*-terminal tail of PKC-ι (Figure 6D). This tail is believed to bring a hydrophobic residue, Phe543 into van der Waals contact with the adenosine head of ATP and the tail forms H-bonds with the glycine-rich loop, thereby ensuring an activated state for PKC-ι [49]. The zinc finger of *Cp*CDPK4 is in the vicinity of the *N*-lobe and the exact volume occupied by the PKC-ι *C*-terminal tail. Instead of a phenylalanine, however, an isoleucine (Ile121) is in position to maintain the more hydrophobic area for the adenosine head group. In addition, for human CLK1 and CLK3 (PDB: 1X57 and 2EU9, respectively), the *C*-terminal lobe bears an insertion between the two sheets β7 and β8, termed the CLK-specific βhp-βhp' hairpin, and renders the substrate docking groove inaccessible (as compared to the substrates of MAPK which are able to access a similar groove) [50]. Although the CLK-specific hairpin partially overlaps with the β-mesh portion of the *Cp*CDPK4 insert, it is distinct in orientation and lacks sequence identity to the *Cp*CDPK4 insert (Figure 6E). Thirdly, although using a deletion mutant to obtain the crystal structure of human SRPK1 that truncates the *N*-terminus by 41 residues and eliminates a spacer of 217 residues (residues 256-473), there is some overlap between the location of the remaining spacer and the *Cp*CDPK4 insert (Figure 6F). There are two small motifs that share sequence identity between the *Cp*CDPK4 insert and SPRK, specifically ^119^TxIxxxP^125 ^and ^128^NExxI^132 ^(numbering from PDB: 3HKO), but only the latter shares spatial and structural similarity, forming the first α-helix in each insert structure but in a nearly orthogonal orientation. For SPRK, the two spacer inserts identified in the structure are necessary for maintaining an active conformation [51]. Although these other inserts have been identified, only further research will determine what particular role both the zinc finger and β-mesh play in the regulation of *Cp*CDPK4. However, the location and other aspects of the β-mesh suggest that it may affect the activation state of this kinase in a specific way. To illustrate, the β-mesh formed downstream of the zinc finger is indeed novel in that its *C*-terminal side forms part of the α-helix D. This helix consists of less than two turns, and at the head features a histidine (His96) that is in H-bonding distance with the alcohol groups of the ribose ring. This position is typically taken up by a glutamate in catalytically active kinases. Additionally, when overlaid with the inactive form of a more prototypical CDPK with the CAD domain (*Tg*CDPK1 [PDB:3KU2]), we can see that the bottom of this β-mesh would interfere sterically with the inhibitory CH1 helix of the CDPK (Figure 6C). All in all, this suggests that the zinc-bound form of this particular CDPK could be constitutively active, as a reversion to the inactive state would be blocked. Attempts to find structures, either active or inactive of the *Cp*CDPK4 with CAD domain intact are underway.

### *Cp*MAP-1 structure

**Figure 7:**
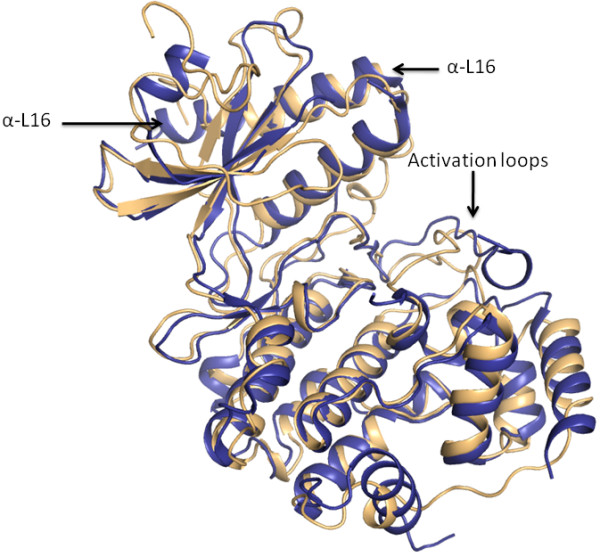
**Comparison of *Cp*MAP-1 with the rat ERK2**. *Cp*MAP-1 [PDB: 3OZ6] is shown in blue, while ERK2 [PDB: 2ERK] is in beige.

The *Cp*MAP-1 structure [PDB: 3OZ6] was solved in the absence of any ATP mimic or inhibitor, and as such the glycine-rich loop, which interacts with β- and γ-phosphates of the ATP, is disordered at the tip in our structure (Figure 7). In comparison with a prototypical MAPK, such as ERK2, the MAPK-specific helix L16 is longer in our structure, and forms more hydrophobic interactions with the α-helix C. The activation loop is moved out of position and no phosphorylation states have been found in the electron density. As is typical with structures lacking ATP/mimic, a good portion of the activation loop is disordered with residues from 175 to 189 absent.

## Conclusions

Overall, protozoan kinases are appealing drug targets, as many are already known to be involved in essential cell cycle regulation; and there is an enormous availability of inhibitors from the extensive work done on human kinase targets. There are several examples of specific kinases to be considered for further investigation with respect to their potential in *C. parvum *drug design, as their orthologues in *Plasmodium *and *Toxoplasma *perform critical functions (see Additional File 1, Table S1 for *C. parvum *orthologues). For example, *Pf*PKG is essential in the blood stage and in gametogenesis of *P. falciparum *infection [22,23], as well, *Tg*PKG is the likely target of an anti-toxoplasmosis compound [52]. *Pf*MRK and *Pf*PK5, respectively, are inhibited by compounds that also modestly inhibit *P. falciparum *growth [34]. TgCK1α is inhibited by aminopurvalanol A which also inhibits parasite cell growth [31]. *Pf*CRK is crucial to intraerythrocytic development [37]. *Pf*MAP-2 is essential to the asexual cycle of *P. falciparum *[40]. *Pf*CK2α has been shown to be crucial to the asexual blood state in *Plasmodium *[44]. Finally, *Pf*TKL-3 is essential for asexual parasite proliferation in human erythrocytes [45]. Other potential *C. parvum *kinase targets to consider include those where there is precedence for preferential inhibition of the parasite kinase (including *P. falciparum *and *T. gondii*) over the human orthologue. For example *Tg*CK1α is selectively inhibited by purvalanol B and aminopurvalanol A over host CK enzymes [31]. As well, a range of compound classes that are CDK inhibitors in *P. falciparum *are not effective against the human CDK enzymes tested [34]. Upon analysis of the kinase sequences of *P. falciparum*, *T. gondii*, and *C. parvum *herein, we find other non-kinase features that should prompt new drug targets to be selected for study. Several *C. parvum *kinases include large (and often multiple) inserts and/or *N*- and *C*-terminal extensions that can be hundreds residues long, but are not conserved and are generally of unknown function. This includes the 40-residue insert in the KD of *Cp*CDPK4 which was structurally identified, herein, as a zinc finger and is suspected to be involved in maintaining its calcium-dependent activity. In addition, there is generally low sequence identity and deviations from the most conserved motifs in the *C. parvum *protein kinases as compared to its orthologues. All of these factors provide additional parameters that can be explored and potentially exploited for drug design not only for cryptosporidiosis, but also for the other protozoan infections.

The high degree of conservation amongst protein kinases, particularly in the ATP-binding site, challenges the general goal of finding selective inhibitors. Nevertheless, the adaptation of particular kinases, for example the CDPK enzymes to specific calcium-binding conditions and other regulatory factors, their localization, and their substrate specificity, adds another dimension to the development of inhibitors, thus mitigating the overall kinase drug discovery process. Our structural and biochemical studies of CDPK enzymes further illuminate some of these possibilities with respect to drug design. The exploitation of the gatekeeper in the ATP-binding site shows that opportunities exist for inhibitor design, as is the case for *Cp*CDPK1 which has a glycine-gatekeeper and is inhibited in the nanomolar range by PP1-derivatives. Indeed, our crystal structure highlights how one of these inhibitors functions. *Cp*CDPK2 structural analysis shows how a flexible CDK inhibitor can bind and gives a starting point for future development of novel CDPK2 inhibitors. *Cp*CDPK4 has a unique zinc finger and special β-mesh configuration that may indicate that it can become persistently active, providing an additional mechanism of regulation to the CaMK family that adds another element to the CDPK drug discovery pathway.

For some time it has been known that the CDPK enzymes control crucial functions including transcription, metabolism, ion pumps and channels, and the cytoskeleton [53]. With respect to the protozoan CDPK enzymes, many have been now associated with particular functions. For example, with respect to *Plasmodium*, *Pf*CDPK1, first identified in the asexual blood stage [54], is involved in the regulation of the motor complex and possibly essential for *P. falciparum *viability [55,56]. *Pf*CDPK3 is implicated in sexual stage specific events [57] and more specifically (for *Pb*CDPK3) in a signalling pathway that regulates ookinete penetration of the layer covering the midgut epithelium and a possibly an ookinete-limited essential function [58,59]. *Pb*CDPK4 is essential for male gametogenesis [60]. *Pf*CDPK5 plays an essential role during the blood stage of malaria replication via egress from erythrocytes [61]. *Pb*CDPK6 is critical for the conversion to an invasive *P. berghei *phenotype [62]. In *T. gondii*, knocking out *Tg*CDPK1 by genetic or chemical means indicates that it is an essential regulator of calcium-dependent exocytosis, specifically leading to the inhibition micronemes secretion that results in a block of essential phenotypes including parasite motility, host-cell invasion, and egress. As well, *Tg*CDPK3 has been suggested to participate in the motility of *T. gondii *through the phosphorylation of glideosome complex member [63]. These results demonstrate the numerous examples of how crucial CDPK enzymes are in both *P. falciparum *and *T. gondii*, and as such suggest that *C. parvum *CDPK enzymes may also be associated with essential functions and should be among the targets of cryptosporidiosis drug discovery programs.

Finally, there are huge untapped kinase sources for drug design, as almost a quarter of the *C. parvum *kinome has no known orthologue outside *of Cryptosporidium spp*. In addition, the OPK group comprises 40% of the *C. parum *kinome, undoubtedly there are unique features within this group to exploit. With numerous kinase inhibitor libraries available, characterization (biochemical and structural) and screening of these kinases may result in the identification of novel targets, potentially without human orthologues, thus greatly facilitating the course of drug discovery. This research can be expedited by considering the kinase classification as presented herein, whereby potential targets are considered not only in the context of their family, but also with respect to their orthologues, a strategy that has streamlined many successful structural genomics projects.

## Methods

### Kinome analysis

To identify protein kinases in the *C. parvum *genome, a search for various protein kinase domains was conducted using the CryptoDB Version 4.3 http://www.cryptodb.org domain search utility [21]. Furthermore, a search for the keyword "kinase" was used. This generated a list of 99 candidates. The presence of a protein kinase domain was confirmed by examining their CryptoDB records, leading to elimination of non-protein kinases, regulatory proteins or other non-kinases. The remaining sequences were analysed manually to confirm the presence of a complete catalytic triad resulting in a final list of 73 kinases. Other protein domains and domain architectures were determined by ProSite http://prosite.expasy.org/[64]. Orthologue group assignments were made by OrthoMCL (Version: 3.0) [65]. The kinase domain sequences of all the *Cp*PKs and the following structures (PDB: 2WEI, 2QG5, 3HKO, 2QKR, 3EB0, and 3OZ6) were submitted for multiple sequence alignment to the PROMALS3D multiple sequence and structure alignment server http://prodata.swmed.edu/promals3d/promals3d.php[66]. The alignment results were slightly adjusted manually in the cases of cgd6_4960, cgd2_2310, cgd7_2000, and cgd2_3890, so that the presumed catalytic lysines were aligned (Additional File 2, Table S2). The adjusted alignment was used in the calculation of the phylogenetic inferences by RAxML BlackBox http://phylobench.vital-it.ch/raxml-bb/index.php[67]. The resulting best scoring ML (maximum-likelihood) tree with branch lengths and support values was submitted to the Interactive Tree of Life Version 2.0.1 website http://itol.embl.de for the rendering of the phylogenetic tree [68]. The same procedure was completed for the analysis of the CDPK family (Additional File 2, Table S3).

### Protein expression and purification

Recombinant samples of *Cp*CDPK1, *Cp*CDPK2, *Cp*CDPK3, and *Cp*CDPK4 (with the following minor truncations (for increased soluble expression): *Cp*CDPK1:M1-E538, *Cp*CDPK2:R186-R667, *Cp*CDPK3:D42-L520, *Cp*CDPK4:L114-R775) were expressed and purified as previously described [27] using entry clones derived from *C. parvum *strain Iowa genomic DNA (from MR4 - Malaria Research and Reference Reagent Resource Center, Manassas, VA, USA), the Lex bioreactor system (Harbinger Biotechnology and Engineering Corp., Toronto, ON) and BL21(DE3)-V2R-pACYC LamP, as the expression host, which includes a plasmid for coexpression of λ-phosphatase to suppress protein phosphorylation.

### Enzymatic characterization and inhibition

Kinase activity was measured using an NADH-coupled ATPase assay (using pyruvate kinase (PK) and lactate dehydrogenase (LDH)) in a 384-well format based on the method of Dölle and Ziegler [69]. For IC_50 _determinations, activities were carried out using 10 nM *Cp*CDPK1, 500 μM ATP, 500 μM Syntide II, and different concentrations of inhibitors (1 nM to 8 μM) in 20 mM Tris, 30 mM NaCl, 10 mM MgCl_2 _1 mM CaCl_2_, 2 μg/ml BSA, 10 mM DTT, and 0.01% Tween 20, pH 7.5. ADP production was measured in BioTek Senergy2 plate reader, using LDH/PK coupled assay with 150 μM NADH, 300 μM PEP and LDH/PK mix from Sigma (with 3 units of LDH/mL). The reaction was initiated with the addition of *Cp*CDPK1. Initial rates were calculated (for 3 to 10 minutes) and data were analyzed using SigmaPlot 9.

### Protein crystallization, data collection, and refinement

Apo *Cp*CDPK1 was crystallized from hanging drops in 0.1 M Tris pH 7.6, 20% PEG 8000, and 0.38 M ammonium sulfate. The crystal was flash-cooled in liquid nitrogen using mother liquor supplemented with 25% ethylene glycol as a cryoprotectant. Data were collected at APS 23ID-B http://www.gmca.anl.gov and processed with XDS indexing and scaling software [70]. *Cp*CDPK1 with the 3MB-PP1 derivative bound was crystallized in sitting drop format from 25% PEG 3350 and 0.1 M BisTris pH 5.5. The crystal was flash-cooled in liquid nitrogen. Data were collected at SLS X10SA http://x10sa.web.psi.ch. Apo *Cp*CDPK2 was crystallized from hanging drops in 2.5 M sodium formate and 0.2 M BisTris propane pH 7.0. Data from the flash-cooled crystal were collected at APS 17ID http://www.sbc.anl.gov and processed with the HKL3000 indexing and scaling software [71]. *Cp*CDPK2 with indirubin E804 bound was crystallized from a solution of 18% PEG 3350, 0.1 M ammonium sulfate, 0.1 M sodium cacodylate and 5 mM indirubin E804. Data for the crystal flash-cooled in liquid nitrogen were collected using a home source Rigaku FRE Superbright rotating anode with an RAXIS IV plate reader. Data were processed using the HKL2000 indexing and scaling software [72]. *Cp*CDPK4 (with 5 mM AMPPNP (phosphoaminophosphonic acid-adenylate ester) and 2 mM MgCl_2_) was crystallized in 0.1 M Hepes pH 7.5, 25% PEG 3350, and 0.2 M NaCl. Data from flash-cooled crystals (with 25% glycerol as a cryoprotectant) were collected on the A1 beamline at CHESS http://www.chess.cornell.edu and processed with HKL2000 [72]. *Cp*MAP-1 crystals formed in 20% PEG 3350, 0.1 M magnesium acetate, 4 mM AMPPNP and 4 mM TCEP-HCl (*tris*(2-carboxyethyl)phosphine hydrochloride salt) by the hanging drop method. Crystals were flash-cooled in liquid nitrogen. Data were collected at APS 19ID http://www.sbc.anl.gov and processed with the HKL3000 indexing and scaling software [71]. Each structure was solved by molecular replacement using modified homology models created with the FFAS03 program [73]. The following models were used: *Cp*CDPK1 used PDB: 2QG5, *Cp*CDPK2 used 1A06, *Cp*CDPK4 used PDB: 3DFA, and *Cp*MAP-1 used PDB: 2B9F. The structures were refined by iterative rounds of manual building in Coot 3.0 and refinement using the ccp4i program refmac5 [74]. All structures were refined with good statistics and geometry, checked with MOLPROBITY [75] and with no outliers in the Ramachandran plot. Final statistics and data information for each structure can be found in Table 4. Figures for structural models were created using the Pymol visualization software http://www.pymol.org and Microsoft PowerPoint.

## Authors' contributions

JDA carried out the kinome analysis, drafted the manuscript, and prepared the figures. AKW analyzed the structural data and wrote part of the structural analysis. AKW, WQ, and VVL collected diffraction data and solved all of the structures (except for [PDB: 2WEI]). AAH, GS, GAW, PF, IC, and MV measured the activity of the CDPK enzymes. YZ and MS also participated in the kinase domain analysis. MA, YL, and AH expressed, purified, and crystallized the CDPK and MAP-1 enzymes. OF and OK screened *Cp*CDPK1 against the inhibitor library. AR and FvD crystallized and solved the *Cp*CDPK1 structure with the PP1 derivative [PDB: 2WEI]. FM, JL, and IK cloned the CDPK and MAP-1 constructs. CZ and KS selected and synthesized PP1 inhibitors. TH analyzed the sequence alignment of CDPK enzymes and identified key motifs. RH conceived the study, initiated the kinome analysis, and helped draft the manuscript. All authors read and approved the final manuscript.

## Supplementary Material

Additional file 1 **Table S1 - Classification of the *C. parvum *protein kinases**. Table listing the 73 protein kinases from *C. parvum*, showing the families, PDB depositions, and orthologues from data compiled from the sequence similarity within the kinase domain, their predicted orthologues, and a maximum likelihood tree from a rapid bootstrap analysis. Click here for file

Additional file 2 **Table S2 - Alignnments of *C. parvum *kinases**. Excel sheet showing the alignment of the 73 protein kinase catalytic domains, as calculated by PROMALS3D multiple sequence and structure alignment server http://prodata.swmed.edu/promals3d/promals3d.php. Table S3 - Alignment of select CaMK enzymes. Excel sheet showing the alignment of the kinase domains of select CaMK family members, as calculated by PROMALS3D multiple sequence and structure alignment server http://prodata.swmed.edu/promals3d/promals3d.php. Click here for file

Additional file 3 **Table S4 - *N*-terminal latch of select CDPK enzymes**. Table showing a partial sequence alignment of CDPK enzymes highlighting conserved *N*-terminal motifs implicated in binding the *N*-terminal latch. Table S5 - Gatekeepers in the *C. parvum *CDPK enzymes. Click here for file
