# Supplementary material for: The Cryptosporidium parvum Kinome
Source: BMC Genomics. 2011 Sep 30;12:478. doi: 10.1186/1471-2164-12-478 (PMC3227725; doi:10.1186/1471-2164-12-478)
Supplement: Additional file 1 — Table S1 - Classification of the C. parvum protein kinases. Table listing the 73 protein kinases from C. parvum, showing the families, PDB depositions, and orthologues from data compiled from the sequence similarity within the kinase domain, their predicted orthologues, and a maximum likelihood tree from a rapid bootstrap analysis. [file 1471-2164-12-478-S1.DOC]

## Table S1 - Classification of the *C. parvum* protein kinases based on the sequence similarity within the kinase domain, their predicted orthologues, and a maximum likelihood tree from a rapid bootstrap analysis.

| *C. parvum* kinase | *C. parvum* structure(s) | Family | *P. falciparum* orthologue | *P. falciparum* annotation | *T. gondii*  orthologue | *T. gondii*  annotation |
| --- | --- | --- | --- | --- | --- | --- |
| cgd1_1220 |  | AGC-  PKA-like |  |  | TGME49_072200 | AGC, PKA |
| cgd2_1830 |  | AGC-  PKA-like | >100 orthologues, but none known from *P.falciparum* or *T.gondii* |  |  |  |
| cgd1_2630 |  | AGC-PDPK | PF11_0227 | AGC | TGME49_068210 | AGC, PDPK |
| cgd3_3040 |  | AGC-PKA | PFI1685w | AGC, PKA | TGME49_026030 | AGC, PKA |
| cgd8_750 |  | AGC-PKG | PF14_0346 | AGC, PKG | TGME49_111360 | AGC, PKG |
| cgd5_2000 |  | Atypical-  RIO | PFL1490w | Atypical, RIO family | TGME49_010830 | RIO1 family domain-containing protein |
| cgd5_2400 |  | Atypical-  ABC1 | PF14_0143 | Atypical, ABC1 | TGME49_046600 | Atypical, ABC1 |
| cgd7_440 |  | Atypical-  RIO | PFD0975w | RIO-like S/T kinase | TGME49_027260 | RIO1 family domain-containing protein |
| cgd8_3250 |  | Atypical-  ABC1 | PF08_0098 | Atypical, ABC1 |  |  |
| cgd6_3400 |  | CaMK | no known orthologues outside of *Cryptosporidium spp.* |  |  |  |
| cgd6_520 |  | CaMK | PFL1885c | PK2 | TGME49_115190 | CaMK, SNF1 |
| cgd7_3890 |  | CaMK | PF14_0476 | protein kinase | TGME49_040630 | ULK kinase |
| cgd2_1060 |  | CaMK-  CDPK2A |  |  | TGME49_006590 | CaMK, CDPK2A |
| cgd2_1300 |  | CaMK-  CDPK5 | PF13_0211 | CaMK, CDPK5 | TGME49_024950 | CaMK, CDPK5, *Tg*PK2 |
| cgd2_1610 |  | CaMK-  CDPK-like | PF11_0060 | CaMK | TGME49_075610 | protein kinase |
| cgd3_260 |  | CaMK-  CDPK | no known orthologues outside of *Cryptosporidium spp.* |  |  |  |
| cgd3_920 | 2WEI ,3DFA,  3IGO, 3MWU, 3NCG | CaMK-  CDPK1 | PF07_0072 | CaMK, CDPK4 | TGME49_101440 | CaMK, CDPK1 |
| cgd4_3330 |  | CaMK-  CDPK6 | PF11_0239 | CaMK, CDPK6 | TGME49_018720 | CaMK, CDPK6 |
| cgd5_2270 |  | CaMK-  CDPK-like | one orthologue from *Thermoplasma volcanium* |  |  |  |
| cgd5_820 | 3LIJ, 3L19 | CaMK-  CDPK3 | PFB0815w | CaMK, CDPK1 | TGME49_105860 | CaMK, CDPK1 (*Tg*PK4) |
| cgd6_650 |  | CaMK-  SNF1 | PF14_0516 | *Pf*KIN | TGME49_033900 | CaMK, SNF1 |
| cgd7_1840 | 2QG5, 3F3Z | CaMK-  CDPK2 | PFC0420w | CaMK, CDPK3 | TGME49_025490 | CaMK, CDPK2, *Tg*PK6 |
| cgd7_40 | 3HKO | CaMK-  CDPK4 | PF07_0072 | CaMK, CDPK4 | TGME49_037890 | CaMK, CDPK4 |
| cgd3_1810 |  | CK1 | PF11_0377 | Casein kinase 1 | TGME49_040640 | CK1α |
| cgd3_40 |  | CK1 |  |  | TGME49_089320 | CK1β |
| cgd1_810 |  | CMGC | no known orthologues outside of *Cryptosporidium spp.* |  |  |  |
| cgd2_3890 |  | CMGC | no known orthologues outside of *Cryptosporidium spp.* |  |  |  |
| cgd7_4850 |  | CMGC | no known orthologues outside of *Cryptosporidium spp.* |  |  |  |
| cgd1_2960 |  | CMGC-  Sky1p-like | PFC0105w | CMGC | TGME49_053440 | CMCG (incomplete catalytic triad) |
| cgd1_60 |  | CMGC-  CDK | no known orthologues outside of *Cryptosporidium spp.* |  |  |  |
| cgd2_1960 | 3OZ6 | CMGC-  MAPK | PF14_0294 | CMGC, mitogen-activated protein kinase 1 |  |  |
| cgd2_4340 |  | CMGC-  MAPK | PF11_0147 | CMGC, MAPK-2 | TGME49_007820 | CMGC, MAPK family |
| cgd3_1510 |  | CMGC-  CDK | no known orthologues outside of *Cryptosporidium spp.* |  |  |  |
| cgd3_3030 |  | CMGC-  MAPK |  |  | TGME49_112570 | CMGC, MAPK family (ERK) *Tg*MAPK-1 |
| cgd4_240 | 3EB0 | CMGC-  GSK | PFC0525c | CMGC, GSK3 | TGME49_065330 | CMGC, GSK family, *Tg*PK3 |
| cgd5_250 |  | CMGC-  Lammer | PF14_0431 | CMGC, *Pf*Lammer | TGME49_024480 | CMGC, Lammer |
| cgd5_2510 | 2QKR, 3NIZ | CMGC-  CDK | MAL13P1.279 | CMGC, PK5 | TGME49_018220 | CMGC kinase, CDK family *Tg*PK2 |
| cgd6_1420 |  | CMGC-  CDK | PFD0740w | CMGC, CDC2-related protein kinase 3 |  |  |
| cgd6_620 |  | CMGC-  CK2 | PF11_0096 | Casein kinase 2, α subunit | TGME49_063070 | CMGC, CK2 family |
| cgd7_1320 |  | CMGC-  CK2 | no known orthologues outside of *Cryptosporidium spp.* |  |  |  |
| cgd7_280 |  | CMGC-  CDK | no known orthologues outside of *Cryptosporidium spp.* |  |  |  |
| cgd7_3050 |  | CMGC-  DYRK |  |  | TGME49_004280 | CMGC, DYRK |
| cgd7_430 |  | CMGC-  CDK | PF10_0141 | MO15-related protein kinase | TGME49_070330 | CaMK, CDPK |
| cgd8_3070 |  | CMGC-  CKL |  |  | TGME49_085160 | CKL-5, putative |
| cgd8_5180 |  | CMGC-  DYRK | PF11_0156 | CMGC | TGME49_113180 | CMGC, DYRK |
| cgd2_3190 |  | OPK-1 | PFC0385c | AGC-related | TGME49_003010 | Aurora kinase |
| cgd3_3180 |  | OPK-1 |  |  | TGME49_035370 | protein kinase |
| cgd6_5060 |  | OPK-1 | PF11_0464 | AGC-related | TGME49_069730 | protein kinase |
| cgd6_5240 |  | OPK-1 | no known orthologues outside of *Cryptosporidium spp.* |  |  |  |
| cgd1_3230 |  | OPK-2 | no known orthologues outside of *Cryptosporidium spp.* |  |  |  |
| cgd6_4960 |  | OPK-2 |  |  | TGME49_043340 | Atypical MEK-related kinase (incomplete catalytic triad) |
| cgd7_1190 |  | OPK-2 | PFC0485w | CaMK | TGME49_018550 | PIK3R4 kinase-related protein |
| cgd7_2000 |  | OPK-2 | no known orthologues outside of *Cryptosporidium spp.* |  |  |  |
| cgd8_1660 |  | OPK-2 | no known orthologues outside of *Cryptosporidium spp.* |  |  |  |
| cgd5_4390 |  | OPK-2-  R45 | MAL8P1.203 | FIKK | TGME49_089050 | FIKk (incomplete catalytic triad) |
| cgd2_2310 |  | OPK-3 | PFI1280c | protein kinase | TGME49_031070 | protein kinase |
| cgd4_1610 |  | OPK-3 | no known orthologues outside of *Cryptosporidium spp.* |  |  |  |
| cgd4_990 |  | OPK-3 | no known orthologues outside of *Cryptosporidium spp.* |  |  |  |
| cgd7_1330 |  | OPK-3 | orthologues, but no other known *Apicomplexan* proteins |  |  |  |
| cgd7_3430 |  | OPK-3 | PFF1145c | protein kinase | TGME49_009050 | TKL |
| cgd8_5120 |  | OPK-3 | orthologues, but no other known *Apicomplexan* proteins |  |  |  |
| cgd1_1490 |  | OPK-4 | PFL1370w | NIMA related kinase 1 |  |  |
| cgd1_890 |  | OPK-4 | PFF1370w | PK4 | TGME49_029630 | protein kinase (incomplete catalytic triad) |
| cgd3_3230 |  | OPK-4 | PF14_0423 | eukaryotic initiation factor 2 alpha kinase 1 |  |  |
| cgd4_3710 |  | OPK-4 | no known orthologues outside of *Cryptosporidium spp.* |  |  |  |
| cgd7_3080 |  | OPK-4 |  |  | TGME49_073690 | Wee kinase |
| cgd8_1230 |  | OPK-4 | PF11_0488 | protein kinase |  |  |
| cgd8_2180 |  | OPK-4 | orthologues (*Trypanosoma*), but no other known *Apicomplexan* proteins |  |  |  |
| cgd7_3760 |  | OPK-4-  NEK | PFE1290w | NIMA related kinase 2 | TGME49_044620 | NEK kinase |
| cgd7_5050 |  | OPK-4-  NIMA | no known orthologues outside of *Cryptosporidium spp.* |  |  |  |
| cgd3_2900 |  | TKL |  |  | TGME49_037210 | TKL |
| cgd8_2430 |  | TKL1 | PFB0520w | TKL1 |  |  |
| cgd3_4310 |  | TKL3 | PF13_0258 | TKL3 | TGME49_053860 | TKL |
