# Supplementary material for: The Cryptosporidium parvum Kinome
Source: BMC Genomics. 2011 Sep 30;12:478. doi: 10.1186/1471-2164-12-478 (PMC3227725; doi:10.1186/1471-2164-12-478)
Supplement: Additional file 3 — Table S4 - N-terminal latch of select CDPK enzymes. Table showing a partial sequence alignment of CDPK enzymes highlighting conserved N-terminal motifs implicated in binding the N-terminal latch. Table S5 - Gatekeepers in the C. parvum CDPK enzymes. [file 1471-2164-12-478-S3.DOC]

**Table S4** - Conserved hydrophobic residues that may be key for the *N*-terminal latch of CDPKs, including those upstream of GxGxxG as well as the two patches of hydrophobic residues, termed key hole 1 and key hole 2 that may complete the closure of the latch.

| CDPK | Classification | latch | key hole 1 | key hole 2 |
| --- | --- | --- | --- | --- |
| TGME49_037890 | ? | PLFTSRIKTK-------VKLEQV**Y** | AIKRASLALIAMSM | LRYVLGDSYDS |
| TGME49_095760 | ? | AFQVCSRLVR-------GSIHKD**Y** | KLRRANLTMMAYSV | LRVLLGDRYSN |
| cgd2_1060 | FxRxxFILxxxG | FRREGLIPACK------GSIHSD**Y** | RFMKVALTVIAQQM | LLNVFSYNSEQ |
| cgd7_1840 | FxRxxFILxxxG | LDRSRFILSTK------GDINQY**Y** | RLKKIALTLIAQNI | FLKVMSLSSKA |
| PFC0420w | FxRxxFILxxxG | FSRRGFILSFT------GNLEDF**Y** | KFQKLAMTIIAQQS | ----------- |
| PFF0520w | FxRxxFILxxxG | E-RKNLILCHS------GKLEDK**Y** | ELKKIALTIIAKHL | LKRIFGRDDIE |
| TGME49_006590 | FxRxxFILxxxG | IDRSKFILENT------GALTDF**Y** | KLKKAALTVIAQHM | LKKVLGMPDVE |
| TGME49_025490 | FxRxxFILxxxG | FKRSAFILANT------GPITNY**Y** | RLKKLALTVIAQHL | LRQVFHMAG-- |
| TGME49_042400 | FxRxxFILxxxG | FRREGFILSYS------GPLTDY**Y** | KLKKAALTVIAQQM | LAQLLVSVDVQ |
| cgd2_1300 | FxRxxooxxxxL | FDRTCLIQEHALVNR---NINDF**Y** | KLKKLALTCVAYHL | LSRVLNITFLQ |
| cgd4_3330 | FxRxxooxxxxL | --RDQFVRRTSL--KKADDIESIF | QLKHALVNMMAHQL | ----EFEKVLC |
| cgd7_40 | FxRxxooxxxxL | FSHNAFVSLGSL-----LELQKK**Y** | LLKKIALTVIAR-Y | IKKLVSSEKIR |
| PF11_0239 | FxRxxooxxxxL | --RNCIISRKKLGG-YDYSSIYN**Y** | NIRNIIINIMAHEL | IVSLVHDKVLD |
| PF11_0242 | FxRxxooxxxxL | --RDEWVQALYS-STKQNTLYNL**Y** | PLHIEEEKNTEEIE | GHTVQINDNTN |
| PF13_0211 | FxRxxooxxxxL | FDRSQIIQEIILMNND--ELSDV**Y** | KIKKLAVTCIAYQL | LFKILSFSAVQ |
| TGME49_018720 | FxRxxooxxxxL | --RSVFVGRRNLGE-SKKTIDDLF | HLKNALVNLMAHQL | ----EFCDLVL |
| TGME49_024950 | FxRxxooxxxxL | FDRSKIVHEVLLKDGQQ--ITDI**Y** | KLKKLAITCVAYQL | LQKVLETRCVQ |
| TGME49_028750 | FxRxxooxxxxL | --REWVDTLRVAT--KQQALEQL**Y** | GLDPGAPTSVATPV | LSPAAGSKSVP |
| TGME49_092060 | FxRxxooxxxxL | LKRQQLICSSTL------PITTC**Y** | ----AASPPSQRPS | ----------- |
| cgd3_920 | xxPGMFIxxxxG | VTTGMFVQSGS------GTFAER**Y** | KLAQAALLYMASKL | LFKLFSQADSS |
| cgd5_820 | xxPGMFIxxxxG | ATPGMFITSKK------GHLSEM**Y** | KLAQAALLYMASKL | LASVFG--LDH |
| PF07_0072 | xxPGMFIxxxxG | LRPGMFIQNSN------VVFNEQ**Y** | KLAQAALLYMGSKL | LAQLFG--LSD |
| PFB0815w | xxPGMFIxxxxG | INPGMYVRKKE------GKIGES**Y** | KLAQAAILFIGSKL | LANLFG--LTS |
| TGME49_101440 | xxPGMFIxxxxG | ATPGMFVQHST------AIFSDR**Y** | KLAQAALLYMGSKL | LATIFG--VSD |
| TGME49_105860 | xxPGMFIxxxxG | MTPGMYITQQK------AHLSDR**Y** | KLAQAAMLFMGSKL | LGRLFG--VTE |

**Table S5** - Partial alignment of *Cp*CDPKs showing the gatekeeper residues in yellow and noting the unique glycine gatekeeper in *Cp*CDPK1.

| ***Cp*CDPK** | **Partial alignment of subdomain II** |
| --- | --- |
| cgd3_260-CDPK | HPNIAKLFESFEDYNSIYLIMELCTGGELFDRL |
| cgd3_920-CDPK1 | HPNIMKLFEILEDSSSFYIVGELYTGGELFDEI |
| cgd7_1840-CDPK2 | HPNIIRLYETFEDNTDIYLVMELCTGGELFERV |
| cgd2_1060-CDPK2A | HPNIVKLYETYQDKENIYLVMELCSGGELFDRI |
| cgd5_820-CDPK3 | HPNIMKLYDFFEDKRNYYLVMECYKGGELFDEI |
| cgd7_40-CDPK4 | HPNIARLYEVYEDEQYICLVMELCHGGHLLDKL |
| cgd2_1300-CDPK5 | HPNIIKLFEVFEDTNYLYFVMEICTGGELFDRI |
| cgd4_3330-CDPK6 | HPNIIKIFEVYEDKEHMYIIMENCGGGELFERI |
| **Sequence Conservation** | ****** :::: :* :: * **.*::.:** |
